# Multi-Omics of Pine Wood Nematode Pathogenicity Associated With Culturable Associated Microbiota Through an Artificial Assembly Approach

**DOI:** 10.3389/fpls.2021.798539

**Published:** 2022-01-03

**Authors:** Shouping Cai, Jiayu Jia, Chenyang He, Liqiong Zeng, Yu Fang, Guowen Qiu, Xiang Lan, Jun Su, Xueyou He

**Affiliations:** ^1^Fujian Academy of Forestry Sciences, Fuzhou, China; ^2^Fujian Provincial Key Laboratory of Haixia Applied Plant Systems Biology, Basic Forestry and Proteomics Research Center, Fujian Agriculture and Forestry University, Fuzhou, China; ^3^College of Forestry, Fujian Agriculture and Forestry University, Fuzhou, China; ^4^Institute of Soil Fertilizer, Fujian Academy of Agricultural Sciences, Fuzhou, China; ^5^Natural Resources Bureau of Shanghang County, Longyan, China

**Keywords:** pine wilt disease, pinewood nematode (*Bursaphelenchus xylophilus*), symbiotic microbiota, metabolome, ROS, transcriptome

## Abstract

Pinewood nematode (PWN), the causal agent of pine wilt disease (PWD), causes massive global losses of Pinus species each year. Bacteria and fungi existing in symbiosis with PWN are closely linked with the pathogenesis of PWD, but the relationship between PWN pathogenicity and the associated microbiota is still ambiguous. This study explored the relationship between microbes and the pathogenicity of PWN by establishing a PWN-associated microbe library, and used this library to generate five artificial PWN–microbe symbiont (APMS) assemblies with gnotobiotic PWNs. The fungal and bacterial communities of different APMSs (the microbiome) were explored by next-generation sequencing. Furthermore, different APMSs were used to inoculate the same Masson pine (*Pinus massoniana*) cultivar, and multi-omics (metabolome, phenomics, and transcriptome) data were obtained to represent the pathogenicity of different APMSs at 14 days post-inoculation (dpi). Significant positive correlations were observed between microbiome and transcriptome or metabolome data, but microbiome data were negatively correlated with the reactive oxygen species (ROS) level in the host. Five response genes, four fungal genera, four bacterial genera, and nineteen induced metabolites were positively correlated with the ROS level, while seven induced metabolites were negatively correlated. To further explore the function of PWN-associated microbes, single genera of functional microbes (Mb1–Mb8) were reloaded onto gnotobiotic PWNs and used to inoculate pine tree seedlings. Three of the genera (*Cladophialophora*, *Ochroconis*, and *Flavobacterium*) decreased the ROS level of the host pine trees, while only one genus (*Penicillium*) significantly increased the ROS level of the host pine tree seedlings. These results demonstrate a clear relationship between associated microbes and the pathogenicity of PWN, and expand the knowledge on the interaction between PWD-induced forest decline and the PWN-associated microbiome.

## Introduction

Pine wilt disease (PWD) is one of the most devastating forest diseases in the world. PWD has increased the number of its host species and its pandemic area rapidly in the last decade, and causes enormous economic losses annually (Proença et al., 2017b,a). The pinewood nematode (PWN), *Bursaphelenchus xylophilus*, is the causative agent of PWD and is transmitted by its insect vector, the *Monochamus* beetle (Zhao et al., 2014). Numerous studies have been conducted to uncover the pathogenic mechanisms of PWD through the exploration of the ecological and genetic connections among hosts, PWN, and the insect vector (Zhao et al., 2014; Espada et al., 2016; Ryoji et al., 2021). Several critical pathogenic factors have been proposed [i.e., the abnormal regulation of reactive oxygen species (ROS) in the host, the genome of PWN, the regulation of metabolites, and the co-evolution of the PWN-insect vector], providing new insights into PWD management (Shinya et al., 2013; Zhao et al., 2016; Wen et al., 2021). It is well documented that abnormal regulation of ROS is the most important early symptom of PWD, which is also highly correlated with the pathogenicity of PWN (Zhang et al., 2019).

Other than PWN and its insect vector, microbial communities associated with PWN play an important role in the pathogenic mechanisms of PWD (Proença et al., 2017b). Plant-associated microbial communities confer multiple beneficial advantages on their host plants, such as pathogen resistance (Muhammad et al., 2021). The endophytic bacteria and fungi of pine trees have been found to be affected by the invasion of PWN, as the diversity of host endophytic microbiota has been found to increase with the severity of PWD (Proença et al., 2017a; Liu et al., 2019). Recently, some endophytic bacterial strains have been selected to induce resistance against PWD by *in vitro* inoculation (Han et al., 2021). In addition, some secondary metabolite-producing endophytic bacteria have proved useful in the management of PWD, such as by having biotechnological potential, producing antibiotics, and acting as biological control agents against PWN (Qin et al., 2011; Proença et al., 2017b). Moreover, there is a growing body of research documenting the functions of microbiota isolated from the PWN cuticle, especially for bacteria (Roriz et al., 2011; Proença et al., 2017b). These PWN–bacteria interactions can be beneficial (a mutualistic relationship), harmful (a parasitic/pathogenic relationship), or neutral through the adjustment of the microbial community or their metabolites. Although a massive amount of evidence indicates that associated bacteria can produce virulence factors, exo-enzymes, and other harmful secondary metabolites to enhance the pathogenicity of other nematodes, there is limited information available about the relationship between bacteria and PWN pathogenicity (Roriz et al., 2011; Wu et al., 2013; Proença et al., 2017b; Xue et al., 2019; Sajnaga et al., 2021). A comprehensive understanding of these biological interactions can facilitate the future management of PWD, such as by using PWN-associated bacteria as novel biocontrol agents of PWN (Proença et al., 2017b; Wang et al., 2017; Ryoji et al., 2021).

The application of high-throughput sequencing has promoted the multi-omics studies of PWN-associated microbes and the underlying pathogenic mechanism, generating masses of data and revealing more complex relationships between PWN and microbes. Concomitantly, the rapid increases in data have magnified the limits of the microbiome and reduced the application of these studies (Xue et al., 2019; Ryoji et al., 2021; Wen et al., 2021). Although there is only limited evidence implying that PWNs display low genetic diversity in geographic locations, PWN–bacteria symbionts isolated from the host in the field have been directly introduced into different hosts for further profiling of different microbiota without considering the genetic diversity of PWN in different hosts, and this has generated artificial backgrounds (Han et al., 2003; Zhao et al., 2003; Wang et al., 2010; Proença et al., 2017b). Furthermore, although multi-omics has obvious advantages in the discovery of in-depth and fundamental connections among massive data, only separate omics data have been collected to date, which have provided fragments of knowledge about host–PWN–microbe symbiont function(s) in PWD epidemics (Muhammad et al., 2021; Ryoji et al., 2021; Wen et al., 2021). Moreover, general obstacles in microbiome studies, like plate-count anomalies and the inability to distinguish a target microbe from its community (Muhammad et al., 2021), still stand between theoretical and applied research on PWD management.

The current study aimed to explore the relationship between microbes and the pathogenicity of PWN by generating artificial assemblies of culturable PWN-associated microbiota (to conquer the plate count anomaly) from different geographic locations (that differ in the severity of PW epidemic) with the same PWN species population, and inoculating these assemblies onto the same Masson pine (*Pinus massoniana*) cultivar to normalize the background of the inoculation. Multi-omics (microbiome, metabolome, phenomics, and transcriptome) data were obtained in the same inoculation assay to examine the correlation of PWN-associated microbiota and the pathogenicity of PWN in-depth. Furthermore, target microbes were isolated and identified by sequencing at the genus level and were re-introduced individually into the pine tree to verify the correlations built by the multi-omics data. This work demonstrated the correlation between culturable PWN-associated microbiota and PWN pathogenicity based on the genetic, metabolic, and physiological pine tree response. In addition, the newly established PWN-associated microbe library provides fundamental sources for the application of PWN-associated microbiota. This study helps elucidate how associated bacteria and fungi affect the pathogenicity of PWN, and provides useful information on the interaction between disease-induced forest decline and the plant microbiome.

## Materials and Methods

### Isolation of Culturable Pinewood Nematode Associated Microbes From the Field

#### Sampling Area

**FIGURE 1:**
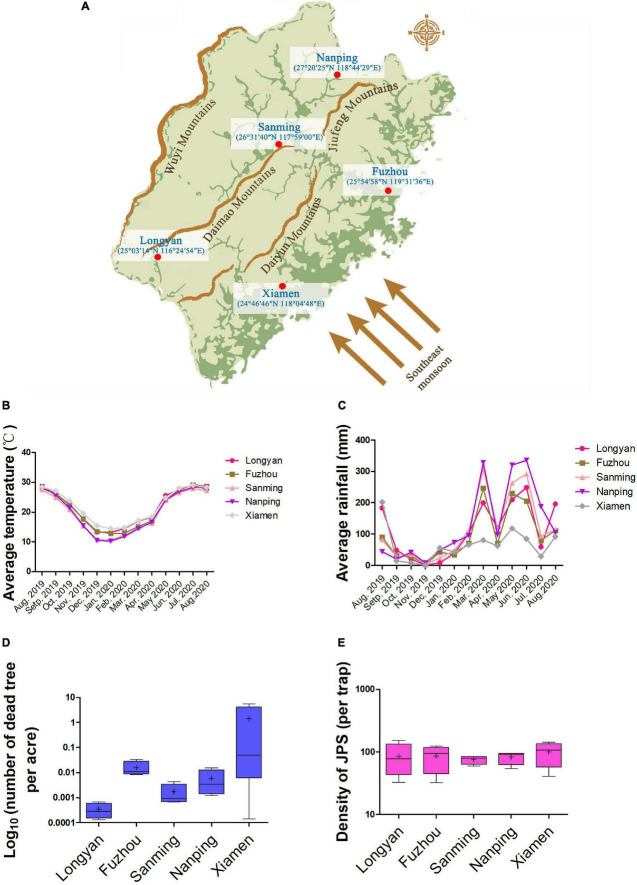
Geographical information and epidemic situation of PWD in sample areas. **(A)** Illumination of basic geographical information of the five sample areas (Fuzhou, Nanping, Sanming, Xiamen, and Longyan). Average temperature **(B)** and rainfall **(C)** were recorded from August 2019 to August 2020. Annual data of fatality rate and density of JPS (Japanese Pine Sawyer) were collected from 2016 to 2020 in the five sample areas and are plotted in **(D)** and **(E)**, respectively. Each box represents the largest and lowest value, with bars representing one standard deviation (SD). “+” represents the mean value.

Sample areas were set in 20-year-old Masson pine (*Pinus massoniana*) forests in five major cities (Fuzhou, Xiamen, Longyan, Nanping, and Sanming) in Fujian, China (Figure 1A). The slope (22°–23°), aspect (120°–125°), and elevation (200–211 m) of the sample areas were similar. Monthly average temperature and rainfall in every sample area were collected from August 2019 to August 2020 by a temperature recorder (LYWSD03MMC, Xiaomi, Shenzhen, China) and rainfall monitor (RS485, Jianda Renke Inc., Shandong, China) (Figures 1B,C). The epidemic situation of PWD from 2016 to 2020 was measured by annual fatality rate data (Figure 1D), and densities of JPS (Japanese Pine Sawyer) were observed (Figure 1E) by APF-1 lure trap (Yongming Biotechnology Inc., Quanzhou, China). The average ratio of JPS that carried PWN among the total trapped JPS was determined by randomly testing five groups of JPS individuals (*n* = 9) for the presence of PWN using PCR (Wu et al., 2021) in Fuzhou (47.8%), Xiamen (48.1%), Longyan (51.6%), Nanping (50.7%), and Sanming (49.2%).

#### Pinewood Nematode Collection and Isolation From the Field

Pinewood nematode (PWN)-infected pine trees (around 30% of needles were red) were chopped into wood pieces (150 cm in length, 15 cm in diameter) from September 1 to 3, 2020, with *n* = 30 trees for each sample area (Fuzhou, Xiamen, Longyan, Nanping, and Sanming). Every 10 wood pieces of each tree that were collected directly from the field and PWNs were isolated using the Baermann funnel method (Han et al., 2010). All PWNs from the same sample area were pooled as one population.

#### Isolation of Culturable Associated Microbes

Pinewood nematodes (PWN) were washed three times with sterilized ddH2O and were transferred into tryptic soy broth (TSB) and a potato dextrose broth (PDB) medium for associated bacteria and fungi isolation, respectively. After incubation at 28°C for 48 h, the associated bacteria and fungi solutions were diluted to 1/10, 1/100, 1/1000, and 1/10000 with TSB, and were then streaked on tryptic soy agar (TSA) and potato dextrose agar (PDA). The plates were incubated at 28°C for 2 weeks, and then single colonies of bacteria and fungi were collected by sample areas and diluted to an optical density (OD600) of 0.15 with sterilized 0.9% NaCl to generate the associated microbe solutions. The single colonies of bacteria on TSA and fungi on PDA plates were harvested separately, and a total of 9,890 bacterial and 7,543 fungal colonies were collected. Microbial colonies were classified into taxonomic genera by sequencing (section “High-throughput microbiome sequencing”). Samples of the different genera were stored in sterilized glycerol solution, with a final concentration of 20%.

### Masson Pine Inoculation Assay

#### Assembly of Pinewood Nematode–Microbe Symbionts

The PWN strain used for further inoculations was isolated from one of the pine trees within the samples from Fuzhou and amplified using a previously described method (Zhu et al., 2012). The PWNs were sterilized by incubation with 1/1000 antibiotics-antimycotic (A6533, Macklin Inc., Shanghai, China) for 4 h, and were then washed three times with sterilized ddH_2_O. The PWNs were transferred to TSA and PDA plates at 28°C for 48 h to check for the presence of any bacterial or fungal colonies. Surface-sterilized PWNs (100,000 individuals per sample) were transferred into the associated microbe or target fungal genus solution and incubated for 24 h at 28°C as reloaded PWNs (in total 14 were made, including 0.9% NaCl control). The reloaded PWNs were maintained on agar plates for 24 h, and were then used for further inoculation as artificial PWN–microbe symbionts (APMSs).

#### Artificial Pinewood Nematode–Microbe Symbionts Inoculation

Two-year-old Masson pine seedlings were maintained in growth rooms (16 h/8 h light/dark cycle) for 2 months before inoculation, with temperatures of 28°C (light), 24°C (dark), and with 70% humidity. Plant infection was performed with 2000 APMSs at 15 cm from the base of each plant, as previously described (Xiang et al., 2015), with *n* = 15 trees for each sample. Sterilized PWNs were used as the control. Validation of inoculation efficiency is shown in Supplementary Figure 1.

### High-Throughput Microbiome Sequencing

#### Sample Preparation

Different APMSs (10,000 individuals for each sample) were collected by centrifuge at a speed of 3000 r⋅min^–1^ for 3 min, and then crushed in liquid nitrogen for further sequencing.

#### DNA Extraction

Total genomic DNA was extracted from samples using the MoBio PowerSoil DNA Isolation Kit (12855-50, MoBio, United States) according to the manufacturer’s protocol. DNA quantity and quality were measured on a NanoDrop 2000 spectrophotometer (Thermo Fisher Scientific, United States). DNA integrity after extraction was determined using 1% agarose gels. Extracted DNA were stored at −80°C until further use.

#### PCR Amplification and High-Throughput Sequencing

The V3 – V4 hypervariable region of bacterial 16S rRNA genes was amplified with the primers 338F (5′-ACTCCTACGGG AGGCAGCAG-3′) and 806R (5′-GGACTACHVGGGTWT CTAAT-3′; H = A/T/C, V = G/A/C, W = A/T). The ITS1-ITS2 hypervariable region of fungal ITS genes were amplified with the primers ITS1 (5′-CTTGGTCATTTAGAGGAAGTAA-3′) and ITS2 (5′-TGCGTTCTTCATCGATGC-3′). PCRs were carried out on a Mastercycler Gradient (Eppendorf, Germany) using 25-μl reaction volumes containing 12.5 μl KAPA 2G Robust Hot Start Ready Mix, 1 μl of forward primer (5 μM), 1 μl of reverse primer (5 μM), 5 μl of DNA (total template quantity was 30 ng), and 5.5 μl of H_2_O. Cycling parameters were 95°C for 5 min, 28 cycles of 95°C for 45 s, 55°C for 50 s, and 72°C for 45 s, followed by a final extension at 72°C for 10 min. Three PCR products per sample were pooled to mitigate reaction-level PCR biases. The PCR products were purified using a QIAquick Gel Extraction Kit (QIAGEN, Germany) and quantified using real-time PCR. Deep sequencing was conducted on a Miseq PE300 platform at Allwegene Company (Beijing, China), and then image analysis, base calling, and error estimation were performed using Illumina Analysis Pipeline Version 2.6.

#### Statistical and Bioinformatics Analyses

The 16S rRNA and ITS gene sequences were processed and demultiplexed through the open-source software pipeline QIIME2 (Version 1.8.0). Quality filtering and trimming, denoising (error-correction), pair-end read merging, chimeric removal, and taxonomy assignments were processed by Vsearch (Version 2.7.1). Analyses were performed using IBM SPSS, version 22.0 (IBM, Chicago, United States).

Clean tags were used to cluster gene sequences (or reduce noise) to generate operational taxonomic units (OTUs). The clustering method could be selected as uparse, uclust, or ref reference database, with the default being uparse; the noise reduction method was the unoise3method (Edgar, 2010, 2013; Rognes et al., 2016). Shannon rarefaction curves and other richness and diversity indices of bacterial community (i.e., Chao1, observed_species, and PD_whole_tree) were estimated using the QIIME2 platform. One-way analysis of variance (ANOVA; Tukey’s test) was performed to determine the differences among groups.

### Metabolome Sequencing

#### Extraction of Metabolites

Fourteen days post-inoculation (dpi), pine tree seedlings (*n* = 5) were harvested and rushed in liquid nitrogen, and then metabolites were extracted following a previously described protocol (De Vos et al., 2007). A sample inoculated with gnotobiotic PWN was set as the negative control for further normalization.

#### Ultra High-Performance Liquid Chromatography-Mass Spectrometry-Mass Spectrometry Analysis

Ultra High-Performance Liquid Chromatography (UHPLC) separation was carried out using an Agilent 1290 Infinity II series UHPLC System (Agilent Technologies) equipped with a Waters ACQUITY UPLC BEH Amide column (100 × 2.1 mm, 1.7 μm) (Dunn et al., 2011). The mobile phase A was 10 mmol/L ammonium formate and 10 mmol/L ammonia, and the mobile phase B was acetonitrile. The elution gradient is shown in Supplementary Table 2. The column temperature was set at 35°C, the auto-sampler temperature was set at 4°C, and the injection volume was 1 μL (Theodoridis et al., 2008; Zhou et al., 2012).

#### Data Analysis

Data for the negative control were trimmed by different samples to produce APMS-induced metabolites. The resulting three-dimensional data, involving the peak number, sample name, and normalized peak area, were fed to the R package metaX for principal component analysis (PCA) and projections to latent structures-discriminate analysis (PLS-DA) (Wen et al., 2017). A one-way ANOVA test using SPSS 20.0 was performed to compare different treatments. In addition, commercial databases, including the Kyoto Encyclopedia of Genes and Genomes (KEGG^1^) and MetaboAnalyst,^2^ were utilized to search for metabolite pathways.^3^

### H_2_O_2_ and O^2–^ Quantification

The APMS-infected Masson pine samples were harvested at 14 dpi. H_2_O_2_ was extracted from the pine needles and measured according to the method of Doulis et al. (1997). O^2–^ was extracted and measured according to Liu’s hydroxylamine method (Liu et al., 2009).

### Genetic Profiling of Pine Tree Responses to Pinewood Nematode Infection

Total RNAs were isolated from infected pine tree seedlings (14 dpi) with Trizol reagent (Invitrogen). Total RNA (1 mg) was reverse transcribed by the PrimeScript™ RT reagent Kit with gDNA Eraser (TaKaRa). Quantitative PCRs were performed using gene-specific primers (included in the expression of unigenes encoding stress responsive pathways, ROS scavenging pathways, terpenoid biosynthesis pathways, and syncytium formation) that mentioned in previous work (Liu et al., 2017), using the Hieff™ qPCR SYBR^®^ Green Master Mix (Low Rox Plus) on a QuantStudio 6 Flex PCR (ABI). The qPCR signals were normalized to that of the reference gene *c60240.graph_c0* (Liu et al., 2017) in pine trees using the ΔCT method (Fan et al., 2013). Biological triplicates with technical duplicates were performed.

## Results

### Diversity and Construction of Pinewood Nematode-Associated Microbes Based on Different Geographical Microbiota

**FIGURE 2:**
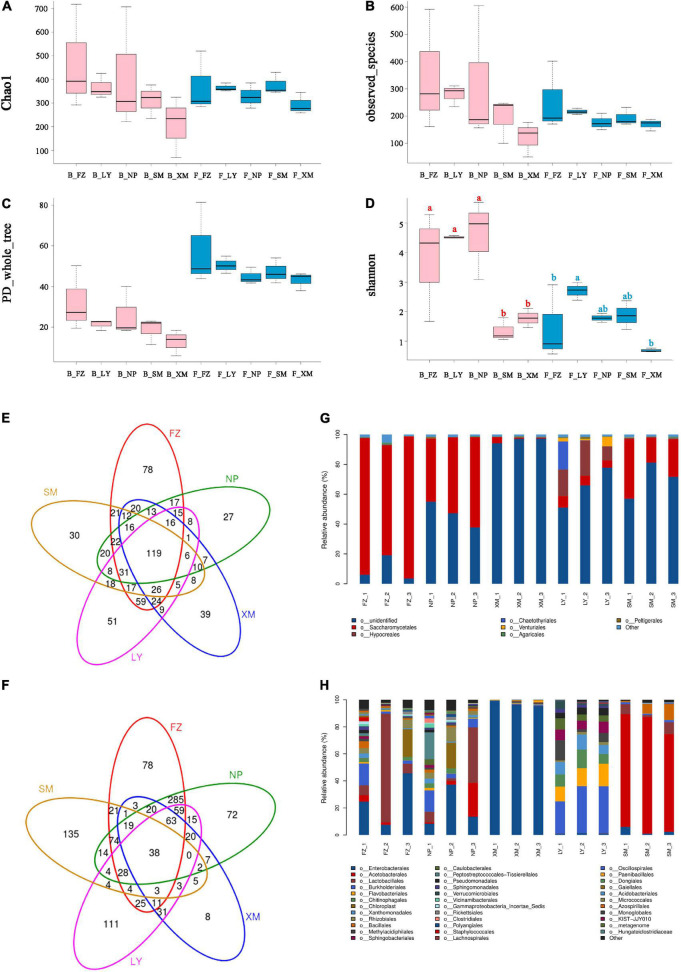
Diversity and construction of associated microbiota in different APMSs. Diversity of associated microbiota in different APMSs were compared and are shown as the following indices: Chao1 **(A)**, observed species **(B)**, PD_whole_tree **(C)**, and Shannon **(D)** of fungal (F_) and bacterial (B_) community in different AMPSs (SM, FZ, NP, LY, and XM indicate the associated microbes originate from Sanming, Fuzhou, Nanping, Longyan, and Xiamen, respectively). Venn diagram of OTU numbers of fungal **(E)** and bacterial **(F)** communities associated with different AMPSs. Construction of fungal **(G)** and bacterial **(H)** communities of different samples are presented at the taxonomic level of order. Different low-case letters (red for bacteria, blue for fungi) above the columns represent significant differences (*P* < 0.05) based on one-way ANOVA, with multiple comparison analysis using Tukey’s test.

In total, 1,290,633 and 944,590 clean reads were collected by ITS and 16S rRNA gene sequencing, respectively. All the associated fungi could be assigned to 753 OTUs, belonging to two kingdoms, 14 phyla, 33 classes, 78 orders, 132 families, 191 genera, and 243 species. The associated bacteria were assigned to 1,163 OTUs, belonging to two kingdoms, 31 phyla, 71 classes, 143 orders, 213 families, 350 genera, and 274 species. Microbial community diversity was measured by the alpha diversity. Both the bacterial and fungal communities of APMSs that developed with microbes from Fuzhou (FZ) had the highest Chao1, observed_species, and PD_whole_tree indices, while the fungal community from the Longyan (LY) sample held the highest Shannon diversity index, and the bacterial community from the Nanping (NP) sample had the highest Shannon diversity index (Figures 2A–D).

There were 78, 27, 39, 51, and 30 specific OTUs in the fungal communities of different APMSs that developed with microbes from FZ, NP, Xiamen (XM), LY, and Sanming (SM), respectively (Figure 2E), while 119 OTUs were shared by all five geographical locations. In the bacterial communities of different APMSs, there were 78, 72, 8, 111, and 135 specific OTUs for FZ, NP, XM, LY and SM, respectively, and 38 OTUs were shared by all five geographical locations (Figure 2F). The fungal communities of the samples from FZ, NP, XM, and SM shared the same dominant order, *Saccharomycetales*, while *Hypocreales* and *Chaetothyriales* were the dominant orders of LY (Figure 2G). For bacterial communities, samples from FZ, NP, and XM shared the same dominant order, *Enterobacterales*, while *Burkholderiales*, *Flavobacteriales*, *Chitinophagales*, and *Chaetothyriales* were the dominant orders of the LY sample, and Acetobacterales was the dominant order of SM (Figure 2H).

### Metabolic Composition Induced in the Hosts by Inoculation With Different Artificial Pinewood Nematode–Microbe Symbionts

**FIGURE 3:**
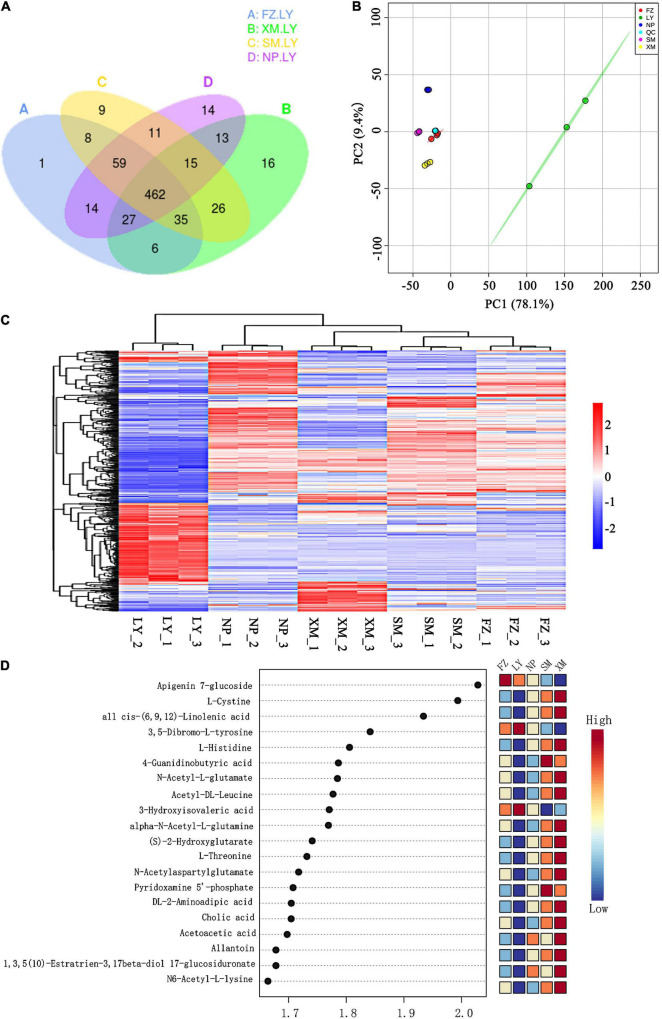
Induced metabolite composition of host plants by different APMSs. **(A)** Venn diagram of induced metabolites for different AMPSs (SM, FZ, NP, LY, and XM indicate the associated microbes originating from Sanming, Fuzhou, Nanping, Longyan, and Xiamen, respectively) compared with that of LY. **(B)** PCA of different AMPS samples. Each point in the figure represents a sample, and different colors represent different groups. **(C)** Hierarchical cluster analysis heat map of all differential metabolites regarding different AMPSs. **(D)** VIP scatter diagram of different metabolites (showing the top 20 differential metabolites based on VIP value). The abscissa is the VIP value calculated by the OPLS-DA model. The color represents the expression pattern of differential metabolites.

In total, 1,301 metabolic substances were induced within different pine tree seedling samples and included 521 commonly induced metabolites (Supplementary Figure 2). No obvious differences were found between FZ, XM, SM, and NP, but they all had specific induced metabolites, unlike LY. Thus, the metabolic composition of FZ, XM, SM, and NP was plotted in comparison with LY, and revealed 132, 119, 151, and 153 specific induced metabolites, respectively (Figure 3A). Principal component analysis of samples (including quality control samples) was employed to preliminarily analyze the overall metabolic differences and variability between samples. The contribution rate of the first principal component was 78.1%, while that of the second was 9.4%, and the sum of the two contributing rates was 87.5%. The PCA demonstrated that LY could be clearly distinguished from SM, NP, XM, and FZ (Figure 3B). Concurrently, the clustering heat map analysis revealed that the composition of induced metabolites was similar within groups. The metabolites induced by the APMS generated with LY samples were different from the other four APMSs, and this was highly correlated with the unique microbial construction of LY APMS compared with the other four APMSs (Figure 3C). LY was significantly higher than FZ, SM, NP and XM in retinol metabolism; tyrosine metabolism; alpha-linolenic acid metabolism; fatty acid biosynthesis; steroid biosynthesis; unsaturated fatty acid biosynthesis; glycine, serine and threonine metabolism; tryptophan metabolism; arginine and proline metabolism; and neuroactive ligand-receptor interaction. XM was significantly higher than FZ, SM, NP, and XM in metabolism pathways in vitamin B6 metabolism, purine metabolism, amino acids biosynthesis, histidine metabolism, and nicotinate and nicotinamide metabolism. The obtained multivariate analysis of the variable importance projection (VIP) of the Orthogonal Partial Least Squares-Discriminant Analysis (OPLS-DA) model facilitated the initial screening of metabolites that differed between different species. Simultaneously, the p-value and fold change (differential fold-change value) of univariate analysis were combined to further screen different metabolites between varieties, and the VIP values of different metabolites showed the top 20 metabolites, with large differences between different species (Figure 3D).

### Associated Microbes Are Crucial to Induce Pinewood Nematode Resistance in Host Plants

**FIGURE 4:**
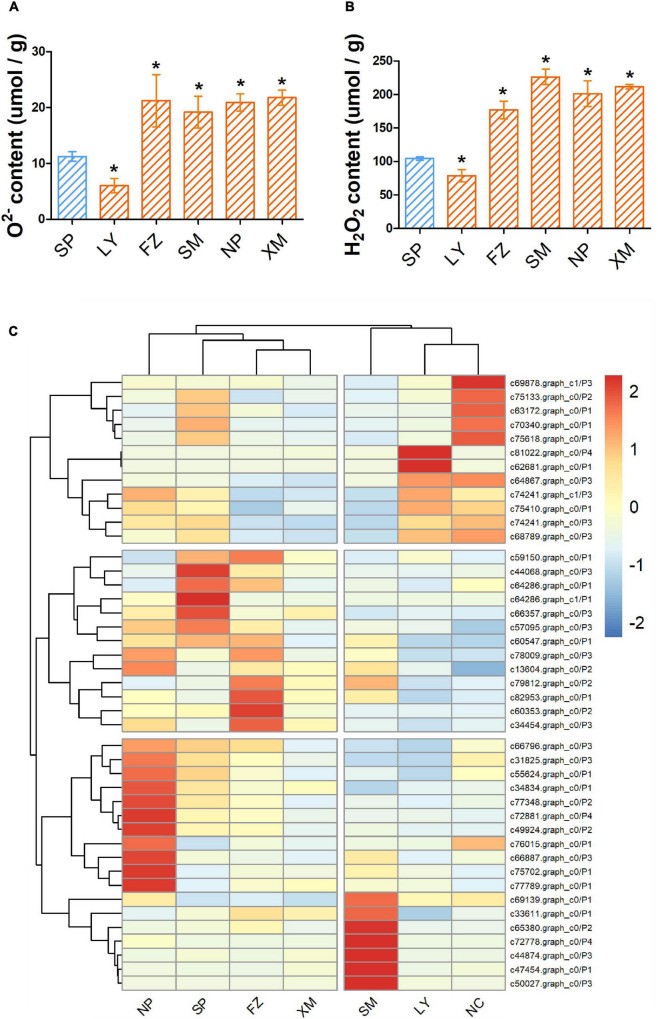
ROS content and the expression of PWN resistance genes in inoculated seedlings. Masson pine seedlings were harvested 14 days after inoculation with different APMSs (LY, FZ, SM, NP, and XM); sterilized PWN (SP) were used as a control. O^2–^ **(A)** and H_2_O_2_ **(B)** content in different samples are represented as mean ± SD. **(C)** Cluster analysis of expression of unigenes encoding stress responsive pathways (P1), ROS scavenging pathways (P2), terpenoid biosynthesis pathways (P3), and syncytium formation (P4) among different samples, with non-infected seedlings as negative control (NC). “*” represents a significant difference compared with SP (Student’s test, *p* < 0.05).

The levels of O^2–^ and H_2_O_2_ (ROS) of different samples were measured at 14 dpi to quantify the pathogenicity of the PWN–microbe symbionts. O^2–^ content in hosts was increased 1.9, 1.7, 1.8, and 1.9 times by NP, SP, FZ, and XM APMSs, respectively, while H_2_O_2_ was increased 1.7, 2.2, 1.9, and 2 times by NP, SP, FZ, and XM APMSs, respectively. However, O^2–^ and H_2_O_2_ contents were reduced to 53.5% and 75.6%, respectively, by LY APMS (Figures 4A,B). In addition, the expression level of PWN-resistance genes related to intergenic in stress response pathways (P1), ROS scavenging pathways (P2), terpenoid biosynthesis pathways (P3), and syncytium formation (P4) were measured. The expression pattern of APMS LY-induced genes was similar to that of the negative control and was highly correlated with the ROS level affected by the LY AMPS (Figure 4C). However, the expression pattern of response genes following invasion with SM AMPS was entirely different to that of LY AMPS. The expression patterns of induced genes in other regions were similar.

**FIGURE 5:**
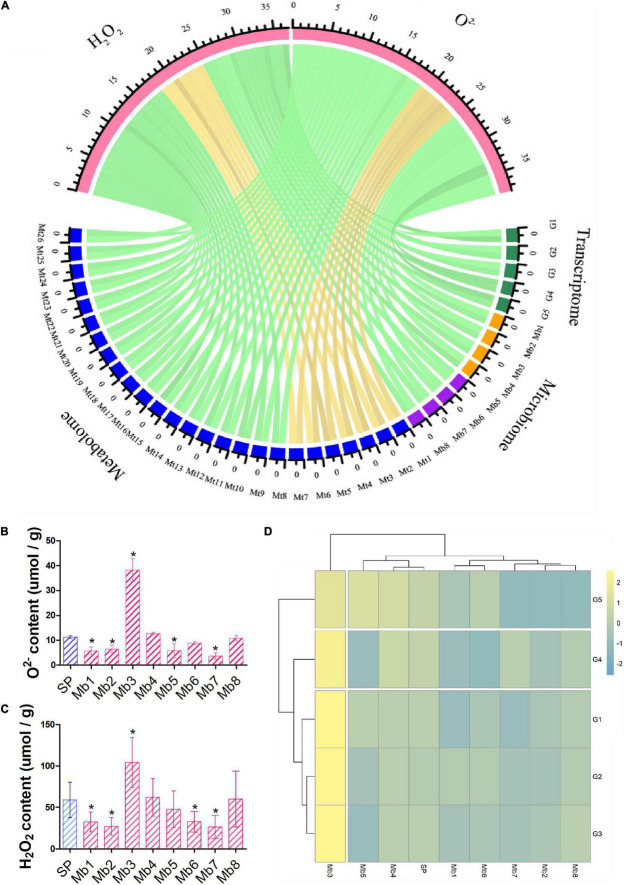
Shortlisting and phenotyping functional microbiota. **(A)** Correlation between phenotypes (H_2_O_2_ and O^2–^) and their highly correlated (*p* < 0.05) transcripts, metabolites, and microbes of different AMPSs are presented as a chord diagram. Gold and green represent positive and negative between samples, respectively. The functional microbes (Mb1–Mb8) were assembled with aseptic PWN and used to inoculate pine trees, and then O^2–^ **(B)** and H_2_O_2_ **(C)** contents and expression level of highly correlated response genes **(D)** in different samples were measured and are represented as mean ± SD. G1–G5 indicate different transcripts (*c64867.graph_c0*, *c81022.graph_c0*, *c62681.graph_c0*, *c68789.graph_c0*, and *c70340. graph_c0*); Mt1–Mt26 indicate different metabolites [3alpha,6alpha-mannotriose, cellobiose, Glu-Gly-Arg, maltotriose, stachyose, thiazolidine-2-carboxylic acid, tyramine, 1,2-di-(9Z-octadecenoyl)-sn-glycero- 3-phosphocholine, 1-hexadecanoyl-2-(9Z-octadecenoyl)-sn-glycero-3-phosphoethanolamine,1-hexadecanoyl-2-octadecadienoyl-sn-glycero-3-phosphocholine, 5-amino-2-methoxyphenol, arachidonic acid (peroxide free), hydroxy-benzene acetic acid, dihydrotachysterol, heptadecanoic acid, hexacosanoic acid, lanosterol, linalool oxide, myo-inositol, nervonic acid, oleoylglycine, non-apropylene glycol, octapropylene glycol, pifithrin-alpha, sterigmatocystin, and uvaol]; and Mb1–Mb8 indicate different microbial genera (*Cladophialophora, Ochroconis, Penicillium, Trichoderma, Achromobacter, Chitinophaga, Flavobacterium*, and *Nubsella*, respectively); SP indicates sterilized PWN. “*” represents a significant difference compared with SP (Student’s test, *p* < 0.05).

Correlation between multi-omics data, measured by Pearson correlation coefficient, showed that the microbiome was positively correlated with the metabolome and transcriptome (*P* < 0.05) and negatively correlated with phenomics (O^2–^ and H2O2), but the correlation with phenomics was not statistically significant (Supplementary Figure 3). Five response genes (*c64867.graph_c0, c81022.graph_c0, c62681.graph_c0, c68789.graph_c0, and c70340. graph_c0*), four genera of fungi (*Cladophialophora, Ochroconis, Penicillium*, and *Trichoderma*), four genera of bacteria (*Achromobacter, Chitinophaga, Flavobacterium*, and *Nubsella*), and nineteen induced metabolites [tyramine,1,2-di-(9Z-octadecenoyl)-sn-glycero-3-phosphocholine,1-hexadecanoyl-2-(9Z-octadeceny)-sn-glycero-3-phosphoethanolamine,1-hexadecanoyl-2-octadecadienoyl-sn-glycero-3-phosphocholine, 5-amino-2-methoxyphenol, Arachidonic acid (peroxide free), hydroxy- benzene acetic acid, dihydrotachysterol, heptadecanoic acid, hexacosanoic acid, lanosterol, linalool oxide, myo-inositol, nervonic acid, oleoylglycine, non-apropylene glycol, octapropylene glycol, pifithrin-alpha, sterigmatocystin, and uvaol] were positively correlated with the ROS level, while seven induced metabolites (3alpha,6alpha-mannotriose, cellobiose, Glu-Gly-Arg, maltotriose, stachyosea, and thiazolidine-2- carboxylic acid) were negatively correlated with the ROS level (*P* < 0.05) (Figure 5A). To further explore the function of PWN-associated microbes, single genera of functional microbes (*Cladophialophora, Ochroconis, Penicillium, Trichoderma, Achromobacter, Chitinophaga, Flavobacterium*, and *Nubsella*) were separately reloaded onto the gnotobiotic PWN and used to inoculate the pine tree. Most of the functional microbes reduced the levels of O2^–^ and H2O2, except *Penicillium*, which significantly increased the level of ROS (Figures 5B,C). Furthermore, colonization with different functional microbes did not activate the expression of PWN response genes in the host, except for *Penicillium* (Figure 5D). Single genera of functional fungi (*Cladophialophora, Ochroconis, Penicillium*, and *Trichoderma*) failed to activate the expression of induced metabolites, while most of the functional bacteria (*Chitinophaga, Flavobacterium*, and *Nubsella*) increased the level of induced metabolites (Supplementary Figure 4).

## Discussion

The functions of PWN-associated microbes are diverse, although most research has focused on their contribution to PWD development (Proença et al., 2017b; Xue et al., 2019). In the current study, PWN-associated microbes from FZ, XM, SM, and NP highly increased the ROS level in the initial stage of PWD (Figures 4A,B), which indicates that the microbes are an important effector of PWD epidemic. However, inoculation of pine tree seedlings with such associated microbes from LY failed to activate the host defense response to PWN, reducing the level of ROS and only weakly altering the genetic response of the host (Figure 4), as well as suppressing anti-toxin genes in the PWN (Supplementary Figure 5). The diversity of fungal community of LY was the highest, and together with the abundance of most associated fungi being positively related to ROS level, this indicated that the diversity of associated fungi of PWN is important to PWN resistance in the pine tree, unlike in the bacterial community (Roriz et al., 2011; Wu et al., 2013; Proença et al., 2017b; Xue et al., 2019). The specific fungi of LY belonging to the order *Hypocreales* has long been identified as an important order of pest control agents (Al Khoury, 2021), which further explains the low pathogenicity of PWN from Longyan. This finding enriches the understanding of microbial agents for PWD control and brings the functional complexity of PWN–microbe symbionts to a new level.

Multi-omics data were collected in this work, and significant correlations were found between the microbiome, metabolome, and transcriptome, but not with phenomics (Supplementary Figure 3). This can be partially explained by the pathogenicity of gnotobiotic PWN and the function of the host endophytic microbiota (Wu et al., 2013; Proença et al., 2017b; Xue et al., 2019). However, although inoculation with a single genus of functional microbe and gnotobiotic PWN significantly affected the ROS level of the host (Figure 5), uncorrelated metabolic, genetic, and phenomics data were collected from most of the samples (Supplementary Figure 4), which indicates that successful induction or suppression of PWD may require intensive interaction between multiple associated microbes and PWN, as well as the host endophytic microbiota. This is also supported by the unstable ability of single microbial agents to control PWNs, which limits the application of microbial nematicide (Zhao et al., 2014; Han et al., 2021). Thus, further investigation is needed.

The successful invasion of PWN increases the diversity of PWN-associated microbiota, but this may result from variations in PWN preference or crosstalk with host microbiota (Roriz et al., 2011; Shinya et al., 2013; Liu et al., 2021). In the current study, co-culture of PWN with culturable microbes from different geographical locations led to a large portion of mutual associated bacterial and fungal microbes (Figure 2). This confirms that PWNs have a preference for particular associated microbes. Furthermore, only a small number of specific metabolites were found in the host after inoculation with four different APMSs (NP, SP, FZ, and XM) (Supplementary Figure 2). This indicates that it is highly likely that the PWN preference of microbes comes from the microbial metabolites, which may also be the foundation of the co-evolution of PWN and its associated microbes (Proença et al., 2017b; Muhammad et al., 2021).

Associated microbes are crucial to both pest damage and plant defenses, gating the co-evolution between host plant and pests in regard to environmental changes (Muhammad et al., 2021). There is increasing evidence of PWN pathogenicity being enhanced by associated bacteria and fungi (Roriz et al., 2011; Shinya et al., 2013; Wu et al., 2013; Proença et al., 2017b), but its contribution to the defense of host pine trees remains unknown. Significant negative correlations were observed between eight genera of microbes and the ROS level, and three of the genera (*Cladophialophora*, *Ochroconis*, and *Flavobacterium*) were able to decrease the ROS level of host pine trees (Figure 5), which was the first report of this function. Among these three genera, one species of *Cladophialophora* (*Cladophialophora chaetospira*) promoted the growth of strawberry plants and suppressed disease severity (strawberry Fusarium wilt) in them (Harsonowati et al., 2020), which hints that species of the genus *Cladophialophora* can reshape components of the microbiota to reduce PWN pathogenicity. In the current study, only one genus (*Penicillium*) significantly increased the ROS level of the host pine tree (Figure 5). However, endophytic species of the genus *Penicillium* were reported to protect their host plant against multiple stresses through their antimicrobial and antioxidant functions (Toghueo and Boyom, 2020), which is contrary to the physiological changes in the host resulting from colonization. Several potential reasons for this paradox should be considered in the future, including reforming the endophytic microbiota of the host and/or PWN-associated microbes by species of the genus *Penicillium*.

The abundance of six genera (*Bovista*, *Inocybe*, *Cyberlindnera*, *Meyerozyma*, *Ogataea*, *Wickerhamomyces*) of bacteria was positively correlated with the ROS level (Supplementary Table 26). These genera belong to the orders of *Agaricales* and *Saccharomycetales*, both of which are known as endosymbionts of insects, with complex enzymatic machineries that can enable their hosts to decompose all plant polymers, including lignin (Urbina et al., 2013; Franck et al., 2016; RuizDueñas et al., 2021). Moreover, the accumulation of seven metabolites corresponded to the increasing of ROS (Figure 5). Four of them (X.3alpha. 6alpha.Mannotriose, Malt triose, Cellobiose, and Stachyose) are products of plant polymer decomposition and are important substrates for the growth of insect intestinal bacteria (Mussatto and Mancilha, 2007; Rotte et al., 2009; Hu et al., 2015). These multi-omics data show that the accumulation of pine tree ROS is beneficial to insects but not PWN, thus modulating the insect feeding behavior, which explains the host preference of the *Monochamus* beetle to those PWN invaded pine trees (Guo et al., 2020). Meanwhile, the rising ROS level led to the accumulation of Tyramine, which can inactivate insect immunoreaction to its parasite and enhance the mobility of nematodes in its hosts (Rotte et al., 2009). This hints that the accumulation of ROS in the host contributes to the transmission of PWN by encouraging the formation of PWN–*Monochamus* beetle symbionts.

This study demonstrated a clear connection between PWN-associated microbes and pathogenicity, and, for the first time, established a culturable PWN-associated microbe library and successfully identified some functional microbial agents that may have applications in PWD management (e.g., currently, we are working on spraying the powder of some functional microbes in the field to serve as PWN control agents). However, the effects of different environmental conditions, host plants, and PWN on these results are ambiguous and require further investigation. In addition, the particularity of the sample from Longyan was highlighted in this work for slowing the PWD epidemic, and some indirect evidence of the microbial control of PWD in this location was provided. However, it is difficult to further conclude any reasonable explanation for this particularity regarding geographical or environmental differences. In our future work, big data related to the multidimensional effectors of the PWD epidemic in Longyan and four other cities will be collected to elucidate the PWD control strategies in Longyan regarding multiple effectors, thus providing more effective large-scale integrated pest management (IPM) strategies in the field.

## Data Availability Statement

The datasets presented in this study can be found in online repositories. The names of the repository/repositories and accession number(s) can be found in the article/Supplementary Material.

## Author Contributions

JS and XH designed the research. SC, JJ, and CH wrote the manuscript. LZ, YF, GQ, and XL performed all analyses and drawings. All authors contributed to the article and approved the submitted version.

## Conflict of Interest

The authors declare that the research was conducted in the absence of any commercial or financial relationships that could be construed as a potential conflict of interest.

## Publisher’s Note

All claims expressed in this article are solely those of the authors and do not necessarily represent those of their affiliated organizations, or those of the publisher, the editors and the reviewers. Any product that may be evaluated in this article, or claim that may be made by its manufacturer, is not guaranteed or endorsed by the publisher.

## Footnotes

1
http://www.kegg.jp

2
http://www.metaboanalyst.ca/

3
https://www.genome.jp/kegg/pathway.html
