# Supplementary material for: Multi-Omics of Pine Wood Nematode Pathogenicity Associated With Culturable Associated Microbiota Through an Artificial Assembly Approach
Source: Front Plant Sci. 2022 Jan 3;12:798539. doi: 10.3389/fpls.2021.798539 (PMC8762061; doi:10.3389/fpls.2021.798539)
Supplement: Supplementary file 3 [file Image_1.PDF]

Supplementary Material

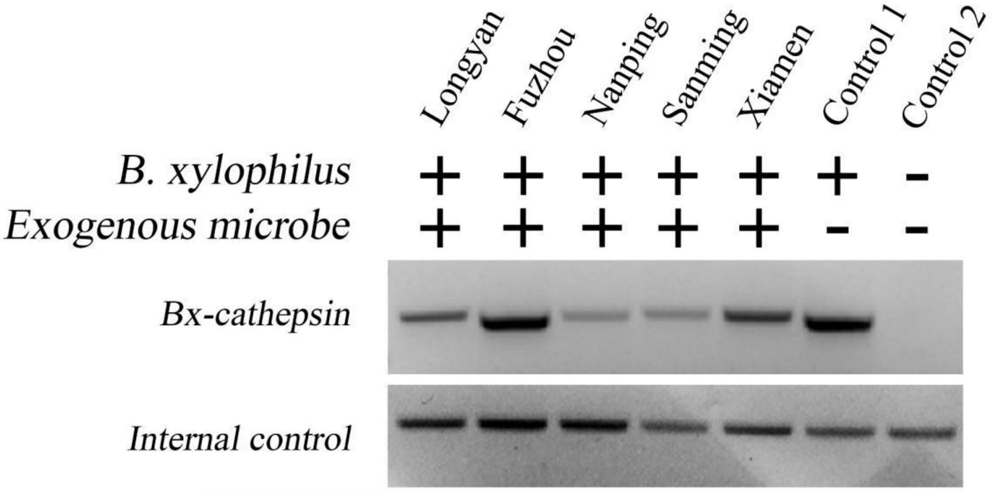

Supplementary Figure 1. Validation of PWN (Pine Wood Nematode) inoculation efficiency. Artificial PWN-microbe symbiont (APMS) that assemble by PWN symbiotic microbes from Longyan, Fuzhou, Nanping, Sanming and Xiamen was inoculated into two-year-old Masson pine seedlings, Sterilized PWN (control 1) and ddH_2O (control 2) were using as control. Plant samples were harvested at 14 dpi (days post-inoculation), DNA was isolated and using as the templates for further validation by PCR reaction. Amplification of Bx-cathepsin (forward primer: 5'-TTGCATTCTACGGCCAGTCC-3'; reverse primer: 5'-ACTGACTTTCGATGGCTCCG-3') represents the valid inoculation, Bx-actin (forward primer: 5'-TTCAGGTGTTACCCACACCG-3'; reverse primer: 5'-GCGGTGGTGGTGAAAGAGTA-3') was using as the internal control to equal the DNA amount.

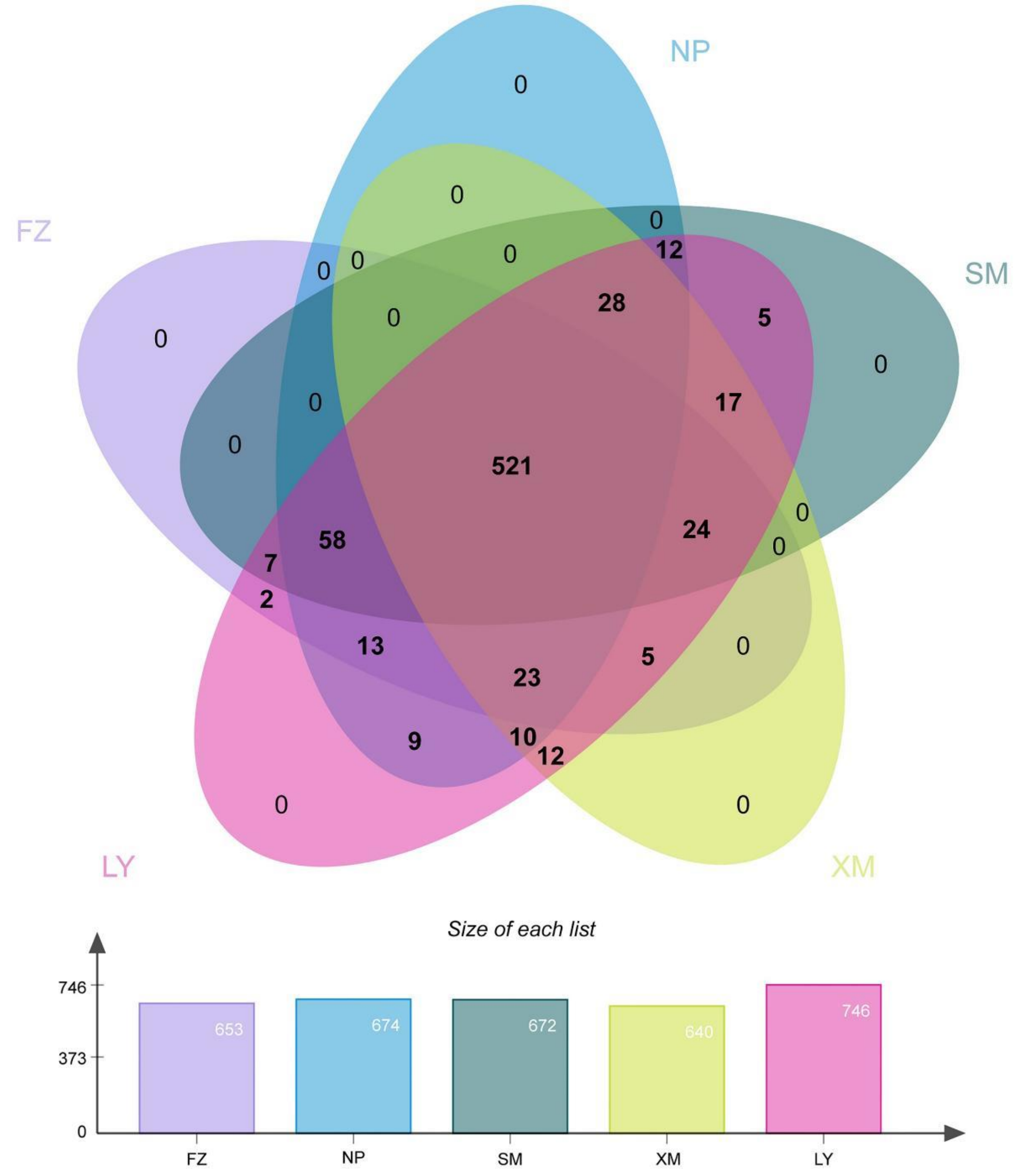

Supplementary Figure 2. Venn diagram of inducing metabolites regard to different AMPSs. (SM, FZ, NP, LY and XM stand for those symbiotic microbes originate from Sanming, Fuzhou, Nanping, Longyan and Xiamen respectively).

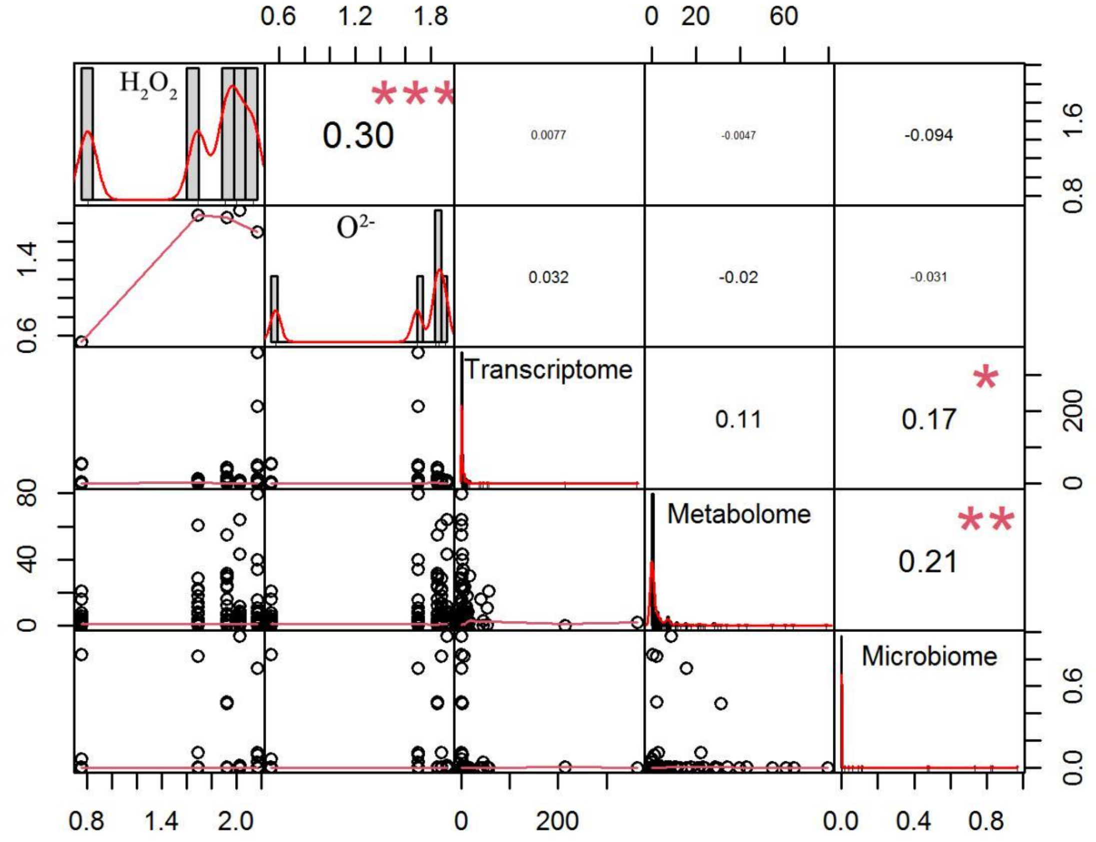

Supplementary Figure 3. Correlation between omics features of different AMPSs. Correlation between features (phenotype (H_2O_2 and O^{2-}), transcriptome, metabolome and microbiome) of different AMPSs is presents by scatter plot. The bar graph represents the data distribution of different features, and the numbers and asterisks represent the specific value and significance of the correlation (*, $0.01 < p < 0.05$; **, $0.001 < p < 0.01$; ***, $p < 0.001$) respectively.

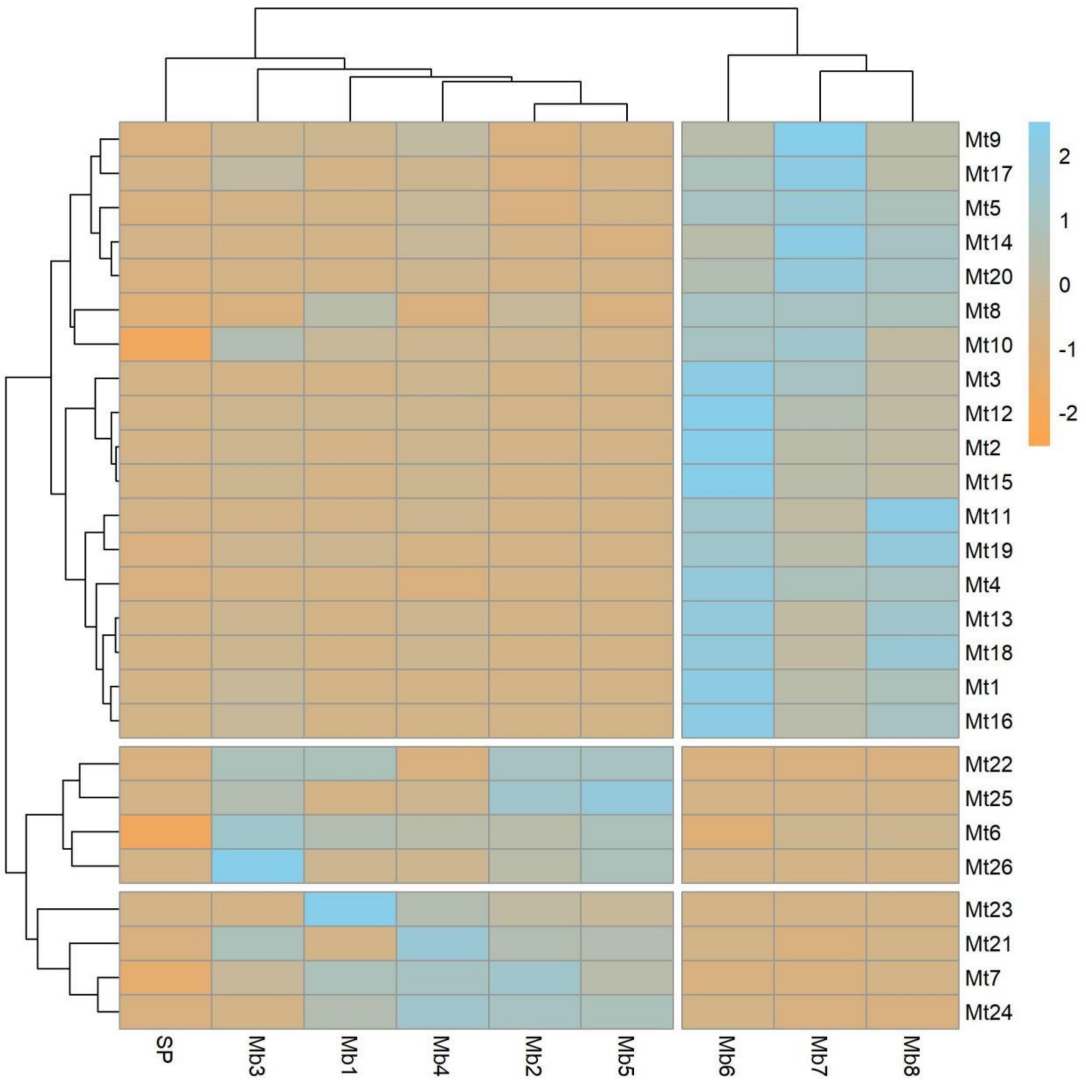

Supplementary Figure 4. Relative abundance of inducing metabolites associates with different APMSs inoculation. On 14dpi of different APMSs (Mb1-Mb8 stand for different microbial genera (Cladophialophora, Ochroconis, Penicillium, Trichoderma, Achromobacter, Chitinophaga, Flavobacterium, Nubsella, respectively) was introduced, 26 inducing metabolites, Mt1-Mt 26 ((3alpha, 6alpha-Mannotriose, Cellobiose, Glu-Gly-Arg, Maltotriose, Stachyose, Thiazolidine-2-carboxylicacid, Tyramine, 1,2-Di-(9Z-octadecenoyl)-sn-glycero-3-phosphocholine, 1-Hexadecanoyl-2 -(9Z-octadecenoyl)-sn-glycero-3-phosphoethanolamine, 1-Hexadecanoyl-2-octadeca dienoyl-sn-glycero-3-phosphocholine, 5-Amino-2- methoxyphenol, Arachidonic Acid (peroxide free), hydroxy-Benzeneacetic acid, Dihydro tachysterol, Heptadecanoic acid, Hexacosanoic acid, Lanosterol, Linalool oxide, myo-Inositol, Nervonic acid, Oleoylglycine, Nonapropylene glycol, Octapropylene glycol, Pifithrin-alpha, Sterig matocystin, Uvaol) , were measured and normalized with the sample of SP (sterilized PWN).

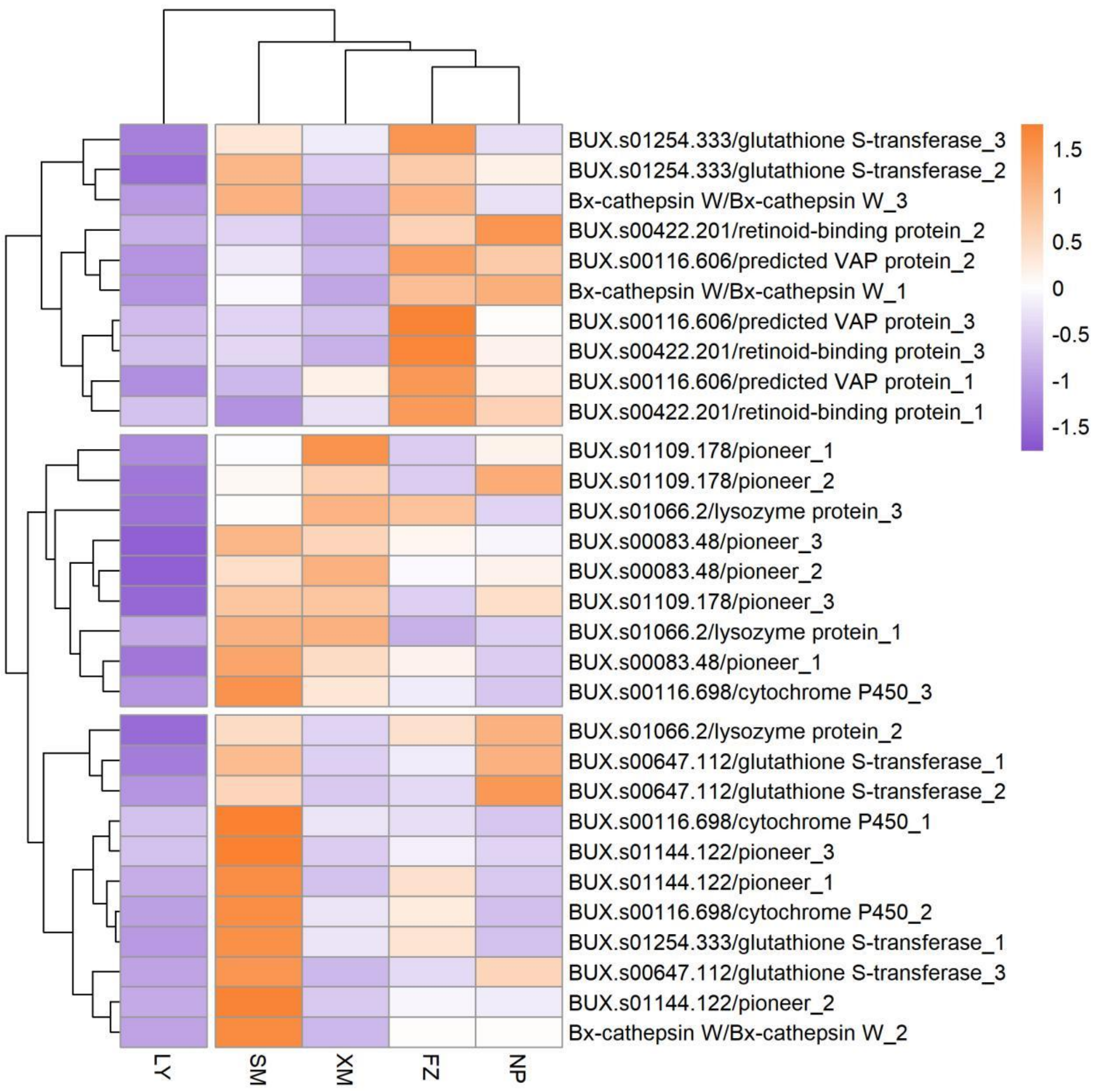

Supplementary Figure 5. The expression pattern of the PWN anti-toxin genes regard to the inoculation of different AMPSs. PWNs were isolated from 14dpi pine tree seedlings and the anti-toxin genes were quantified by RT-qPCR. Heat map drawn in different colors, red, purple and white indicate positive, negative and irrelevant correlations between samples, respectively.
